# A Robust, High-Content NAM for Repeatable and Predictive Developmental and Reproductive Toxicity Assessment in C. elegans

**DOI:** 10.21203/rs.3.rs-9004834/v1

**Published:** 2026-04-10

**Authors:** Sudip Mondal, Adam Laing, Amber Shen, Evan Hegarty, Abhishri Medewar, Sebastian Gomez, Gina Carrion, Julia Brown, Adela Ben-Yakar

**Affiliations:** vivoVerse, LLC; vivoVerse, LLC; vivoVerse, LLC; vivoVerse, LLC; vivoVerse, LLC; vivoVerse, LLC; vivoVerse, LLC; vivoVerse, LLC; The University of Texas at Austin

**Keywords:** Developmental and reproductive toxicology (DART), C. elegans, in utero embryo phenotype, high-resolution imaging, microfluidic technology, standard operating procedure (SOP), repeatable assays, acceptance criteria, new approach methodology (NAM)

## Abstract

Developmental and Reproductive Toxicity (DART) assessment is essential for product safety evaluation and currently relies heavily on vertebrate models that are costly, time-consuming, and resource-intensive. *Caenorhabditis elegans* has emerged as a promising New Approach Methodology (NAM) for rapid, cost-effective, whole-organism toxicology studies. However, broader adoption has been limited by the lack of high-resolution, rapid imaging approaches, a limited range of endpoints, and insufficient evidence for assay robustness and repeatability. To overcome these limitations, we developed a multiparametric imaging-based assay for assessing DART-related endpoints in *C. elegans*, building on our previously published microfluidic-based developmental toxicity platform. We expanded the machine learning-based body dimension analysis to include quantification of total embryo number and *in utero* embryonic development by classifying embryos as early- or late-stage to assess reproductive health. The assay relies on high-resolution brightfield imaging, enabled by the vivoChip microfluidic device, and is compatible with any strain. This study demonstrates highly repeatable results with mean coefficients of variation of 1–5% for developmental endpoints and 6–17% for reproductive-related endpoints, supporting high statistical power. Validation using methylmercury and propiconazole and by phenotyping (scoring and classification) ~ 400,000 embryos across ~ 9,200 worms, demonstrated reproducible, concentration-dependent responses across all endpoints with narrow confidence intervals. Notably, late-stage embryos were the most sensitive endpoint, with effects preceding changes in total embryo count, body size, or viability. Importantly, worms remained > 97% viable and motile across all tested concentrations, indicating that the observed effects reflect DART-specific responses rather than non-specific apical endpoints such as lethality. These results demonstrate a sensitive, repeatable, and scalable DART platform capable of rapid, cost-effective chemical prioritization for safety assessment.

## Introduction

Developmental and reproductive toxicity (DART) assessment is a vital part of product safety testing for chemicals, consumer products, and pharmaceuticals. Currently, most DART testing is carried out in mammalian models such as rats and rabbits for human health risk assessment [1, 2]. However, increased regulatory and societal pressure to reduce animal use has led to new legislative initiatives in the US, EU, and other regions aimed at limiting reliance on vertebrate models. In addition to ethical considerations, mammalian-based DART studies are costly and time-intensive. For example, a standard DART study with rats may require 2–3 months of exposure and monitoring due to the long life cycles, substantial housing infrastructures, and complex experimental design. High costs also restrict sample size, potentially limiting statistical power. Despite their high genetic and physiological similarities to humans, rodent data alone are only 43% predictive of human toxicity outcomes [3]. The concordance improves to 71% when combined with a non-rodent species, supporting a multi-tier approach. With over 350,000 registered chemicals requiring new or updated safety evaluation [4], there is an urgent need for New Alternative Methods (NAMs) to complement or replace traditional methods.

NAM-based DART approaches include *in silico* prediction models [5], *in vitro* systems [6–8], *ex vivo* technologies [9], and alternative invertebrate model organisms not subject to welfare regulations, such as *C. elegans* [10–29], earthworm [30], brine shrimp [31, 32], and *Daphnia* [33, 34]. Although no single method can fully recapitulate human biology, a multi-tiered approach that integrates these complementary models can provide a more comprehensive and human-relevant toxicity profile for risk assessment. *In silico* methods remain constrained by limited, high-quality training datasets [35]. *In vitro* and *ex vivo* systems are highly variable and often capture only isolated adverse outcome pathways (AOPs) and lack the integrated tissue- and organ-level interactions required for complex DART responses. Among invertebrates, standardized DART assessment protocols exist for *Daphnia* and earthworms and are widely used for ecotoxicology risk assessment [34, 36, 37]. Although no regulatory test guidelines currently exist for *C. elegans*, the small model organism has been extensively used in scientific research, including numerous toxicology studies [11–29].

*C. elegans* is a powerful, high-throughput model organism that can be grown quickly and inexpensively in large numbers using well-established techniques. Its small size and simple, transparent body make it highly amenable to imaging-based methods for high-content analysis. The *C. elegans* genome shares extensive conservation with mammals, with up to 60–80% and ~ 83% homology to the human genome and proteome, respectively [38, 39]. It has complete neuromuscular, reproductive, and digestive systems with conserved signaling and metabolic pathways, enabling system-level AOPs studies. The reproductive system of a *C. elegans* hermaphrodite consists of a germline, somatic gonad, uterus, and egg-laying circuitry [40, 41]. Many fundamental biological processes are conserved across species and are similarly preserved in *C. elegans*. Notably, conserved evolutionary mechanisms governing reproduction include germ cell proliferation and differentiation, oogenesis, programmed cell death, chromosomal aneuploidy, and reproductive aging. In addition, *C. elegans* contains at least 76 cytochrome P450 genes [42], Phase I-III xenobiotic metabolism pathways [14], and a functional microbiome, supporting toxicological evaluation of parent compounds and metabolites with unique tissue-level bioavailability profiles [14, 21, 29].

Previous large-scale studies show concordance between *C. elegans* and mammalian toxicity outcomes. Developmental toxicity endpoints demonstrate ~ 53% balanced accuracy for predicting mammalian toxicity [13]. Germline integrity studies analyzing chromosome segregation defects in *C. elegans* embryos achieved 69% balanced accuracy in predicting mammalian reproductive toxicity [23]. Acute toxicity studies of 17 non-acidic chemicals reported 88.5% concordance with rat lethal dose predictions, outperforming mouse–rat concordance (87.9%) [43]. However, many of these studies rely on low-throughput, labor-intensive methodologies or low-resolution information to assess gross developmental defects and embryo fates upon chemical exposures [44, 45], limiting scalability and reproducibility. Advances in microfluidics [46–51] and machine learning (ML)-assisted image analysis [46] now enable robust, high-throughput, and multi-parametric analysis of *C. elegans*.

Here, we present a high-content imaging-based assay to quantify DART-related endpoints in *C. elegans* using an automated high-throughput microfluidic platform that is amenable to automation and large chemical library screening [46–48]. Our assay can be completed within 3 days from initial worm plating and chemical treatment to endpoint imaging. All readouts are derived from brightfield image analysis and are compatible with wild-type strain, transgenic lines, and genetically diverse wild isolates. High-resolution imaging of the entire uterus in 3D enables identification and classification of every *in utero* embryo, providing a more sensitive measure of toxicity than body size alone. Importantly, *in utero* analysis of embryo development during early adulthood is unaffected by the confounding effects from age-associated decline in the reproductive health and off-target effects such as altered egg-laying behavior. Using 30 replicates (5 biological × 6 technical replicates), we show that the DART assay is highly robust and produces repeatable results across multiple phenotypes, extended time periods, and independent of operators. As a case study, we evaluated methylmercury and propiconazole, chemicals with well-characterized DART profiles in *Daphnia*, rodents, amphibians, and fish, to benchmark assay sensitivity and assess translational relevance.

## Materials and methods

### Strains and maintenance

We used the N2 wild type strain (The *Caenorhabditis* Natural Diversity Resource, CaeNDR) for all experiments. *C. elegans* were maintained according to standard methods on nematode growth media (NGM) agar plates seeded with HB101 *Escherichia coli* bacteria at 20°C [52]. Frozen stocks of strains were stored at −80°C and thawed annually to generate a new set of Dauer plates. Sealed Dauer plates were stored at 16°C and used to start fresh maintenance cultures every 3 months by chunking, minimizing genetic drift. We cultured worms for > 5 generations before using them in assays in a temperature and humidity-controlled environment.

### Food preparation

We streaked HB101 from − 80°C glycerol stock onto Luria-Bertani (LB) agar plates with streptomycin and incubated them overnight at 37°C to generate single colonies. A single colony was picked and used to inoculate an overnight 5 mL starter culture in LB broth containing streptomycin. To prepare the HB101 food stock, 200 μl of this starter culture was added to 200 mL LB broth and streptomycin in a 1,000 mL baffled flask. The flask was incubated for 16 hours with shaking at 180 rotations per min (rpm) to reach the late log growth phase. We measured the OD_600_ using a spectrophotometer (DiluPhotometer^™^, Implen) and stored the culture at 4°C. Streak plates and starter or food stocks were used within 2 months and 2 weeks, respectively. Immediately before worm culture, we centrifuged a sufficient volume of the bacterial culture to generate the required volume of OD_600_ = 3.0 food suspension. The LB supernatant was removed, and the bacterial pellet was resuspended in S media. This density was optimal for growing 80–100 larval 1 (L1) to the day 1 (D1) adult stage in a 500 μl culture volume, with sufficient food still remaining after 72 hours to avoid starvation in the worm population prior to DART analysis.

### Chemicals

Methylmercury (II) hydroxide (Cat# 13395, Alfa Aesar, CAS# 1184–57-2, Batch# M25H004) and propiconazole (Cat# 45642–250MG, Sigma Aldrich, CAS# 60207–90-1, Batch# BCCD0480) were dissolved in dimethyl sulfoxide (DMSO, Cat# D2650, Sigma Aldrich, CAS# 67–68-5, Batch# RNBL9635) to prepare master stocks at 100× the highest treatment concentration. The master stocks were transferred into 0.2 ml tubes as single-use aliquots and kept at −80°C until use. For the concentration-response assays, chemicals were serially diluted in DMSO immediately prior to treatment to generate 100× working dilutions, ensuing a final DMSO concentration of 1.0% (v/v) in each well. Solvent control wells received DMSO at the same final concentration (1.0% v/v).

### Worm synchronization and large-scale culture

To generate large numbers of synchronized L1-stage larvae, two NGM plates were each seeded with four parent larval 4 (P_0_ L4) worms. The worms were grown for 48 hours to allow P_0_ to become adults and lay a large number of eggs. At this time, all 8 P_0_ adults (2 NGM plates × 4 adults/plate) were removed. The plates were incubated for an additional 48 hours, by which point the majority of F_1_ generation had reached adulthood. Adult F_1_ worms were collected by washing both plates with M9 buffer and treated with an alkaline sodium hypochlorite solution with alternating low- and high-speed shaking on a speed-controllable vortexer to fragment their bodies and release embryos. The bleaching solution was neutralized with 5 washes of M9 buffer. Embryos were allowed to develop and hatch in M9 buffer in a rotating glass conical tube for 24 hours. Hatched synchronized L1s were filtered through a 20 μm cell filter to remove unhatched eggs and debris. The total number of larvae was counted, and the suspension volume was adjusted to make a final density of ~ 100 L1s/20 μl of M9 buffer.

### Chemical treatment in liquid culture

The age-synchronized L1 suspension (~ 100 larvae in 20 μl volumes) was dispensed into individual wells of a standard 24-well plate with 475 μl HB101 suspension (OD_600_ = 3.0) prepared in S media [53]. Chemicals dissolved in DMSO (5 μl) were added to the designated wells to achieve the desired treatment conditions while keeping a constant solvent concentration across all wells. Vehicle control wells received 5 μl of DMSO solvent (1% v/v). The 24-well culture plates were sealed with airtight films (Cat# 232702, Thermo Scientific) to prevent evaporation and cross-contamination and cultured at 20°C for 72 hours. The plates were shaken every 6 hours using an automated shaking platform (Teleshake 95, Inheco) to homogenize the well contents, including the HB101 distribution, shed cuticles from molting, and the intestinal discharge from the defecation cycles over 24 hours.

### Range-finding assay and live worm quantification

For each chemical tested, we first performed a range-finding assay using at least 20 concentrations (0.009–90 μM for methylmercury and 0.01734–1730 μM for propiconazole) based on published work. Age-synchronized L1s were exposed to different chemical concentrations for 72 hours to determine the maximum soluble concentration in culture medium and assess any significant lethality or growth arrest at the L1 stage. Age-synchronized L1s were exposed to these concentrations in 24-well plates for 72 hours, after which culture plates were imaged and manually analyzed prior to chip transfer. The plate images were used to identify the percentage of live worms and to identify the highest concentration at which the worms developed beyond the L4 stage, ensuring suitability for subsequent vivoChip loading and high-resolution imaging for DART phenotyping.

Specifically, prior to chip imaging, a single-frame and timelapse images (10 s at 10 fps with a 2×, 0.08 NA objective) were acquired from each 24-well culture plate using the vivoScreen system (described below), generating ~ 10.8 GB of data per plate. Single-frame images were inspected to ensure that control wells developed to D1 adult stage according to our predefined assay acceptance criteria (**Supplementary Table 1**). Time-lapse images were inspected to count all worms within the 10,744 × 6,290 μm^2^ field of view (FOV), and spontaneous movement in liquid culture was used as the criterion for viability. The percentage of moving (alive) worms per well was then calculated from 3 biological replicates on different days.

### vivoChip-24x design and operation

Microfluidic-based *C. elegans* immobilization chips (vivoChip-24x, vivoVerse), fabricated from a transparent, low-chemical-absorptivity plastic with broad optical transmission spectrum [21], were used in this study. The device consists of three bonded layers: (i) a top layer containing 24 rectangular wells arranged with 9 mm spacing for compatibility with standard 24-well plates, (ii) an intermediate microfluidic layer containing trapping channels, and (iii) an ultra-thin ~ 80 μm bottom substrate layer enabling high-resolution optical access.

Each well has a loading capacity of 250 μL and is fluidically connected at its bottom surface to an array of 40 parallel tapering microfluidic channels, as previously described [46, 47]. In total, the chip contains 960 channels that orient and immobilize ~ 40 *C. elegans* per well, enabling rapid, high-resolution imaging of 24 distinct worm populations. The channels possess a 3D tapering geometry with an aspect ratio (width to height ratio) close to 1.0, promoting lateral worm orientation. Gradual reduction in channel dimensions allows size-dependent trapping along defined channel regions.

Two vivoChip-24x designs were used: the vivoChip-24x-D1 design with larger channel geometry (tapering from 98 μm × 102 μm at the channel entrance to 24 μm × 40 μm at the exit) and vivoChip-24x-L4 design based on narrower geometry (tapering further to 10 μm × 17 μm at the narrowest region). The D1 design is optimized to trap day 1 (D1) adult worms containing 2–6 *in utero* embryos at the narrowest channel region while allowing laid eggs to pass through. The L4 design enables trapping smaller worms, including early L4 stage worms, and is better suited for populations with fewer or no embryos, although it carries a higher risk of blockage from laid eggs and clogging the microfluidic channels.

Accordingly, two functional designs were used: the vivoChip-24x-D1 (based on the larger channel geometry) and the vivoChip-24x-L4 (based on the narrower geometry). The D1 chip was preferentially used because its wider channels prevented blockage by laid eggs and therefore had a greater number of channels containing trapped worms. The L4 chip was mainly used for worms treated with high concentrations of chemicals that slowed development, resulting in reduced body size and fewer or no *in utero* embryos.

Following removal of the culture plate seal, worms were transferred from each culture plate to the corresponding vivoChip well using a multichannel pipette. Since vivoChip-24x-L4 can trap L4-stage or larger worms, wells containing populations younger than L4 were filled with molten 2% agarose gel to block the channels in those specific wells. The agar block prevented continuous media flow through the open channels and stabilized pressure across all wells during vivoChip imaging.

The chip was placed in a custom vivoChip holder with a sealing gasket to provide controlled fluidic pressurization, as previously described [22]. The vivoChip holder carrying the vivoChip was placed on the microscope stage for imaging and flow was initiated using our vivoCube+ microfluidic control system. Ten on/off pressure cycles were applied, resulting in complete immobilization of *C. elegans* across all 960 channels within ~ 3 minutes.

### Automated image acquisition hardware

The vivoScreen system comprises integrated image acquisition hardware, custom software modules (vivoImager), a centralized searchable Postgres SQL database, and hybrid in-house and cloud-based data storage (**Supplementary Fig. 1**). The image acquisition hardware consists of a customized large FOV inverted microscope (IX73, Evident), a precision *xyz* motorized stage (MS2000, Applied Scientific Instrumentation), a scientific CMOS camera (IRIS-15, Teledyne), and bright light sources (TLED, Sutter Instrument). The large-FOV setup enables imaging of the entire chip area with a 2×, 0.08 NA objective and 8 microfluidic channels simultaneously using a 10×, 0.4 NA objective, capturing all 40 channels with only 5 FOVs. The 10× objective produces a 2.15 × 1.26 mm^2^ FOV, sufficient to capture the full lengths of adult worms across 8 channels with a lateral pixel resolution of 0.425 μm. For DART analysis, volumetric brightfield imaging was performed using 18 z-slices at 6 μm step size to capture the entire worm volume. According to the Nyquist sampling criterion, the theoretical axial resolution for a 0.4 NA objective is 6.5 μm, corresponding to a recommended z-step of 3.25 μm. However, to maintain volumetric imaging speed ~ 1 volume per second with our data readout speed, we used a 6 μm step size. We captured five timelapse 3D hyperstack images at 1-s intervals to analyze twitching in the late-stage embryos if needed.

### Automated image acquisition software and process

To acquire the imaging data, we used custom-developed image acquisition software (vivoImager, **Supplementary Fig. 2**). We captured 10×, 0.4 NA 3D brightfield stacks (18 z-slices) of all worms, centered around the best focal plane of a fiduciary marker (**Supplementary Fig. 3A**). Each vivoChip-24x well contains a cross-shaped fiduciary marker, integrated into the microfluidic channel layer. This feature enables automatic focal plane identification using an edge-detection algorithm (**Supplementary Fig. 3B**). Initially, the software loads a configuration file containing experimental metadata (experiment name, strain name, developmental stage, chemical name, concentration, solvent, etc.) and imaging parameters (illumination intensity, filter sets, camera settings, stage locations, etc.). Then the software initiates all hardware components, calibrates the stage for translation range, and positions the stage at the cross-shaped fiduciary marker of the reference well (B02). Using a pre-defined chip map, the system sequentially navigates to the fiduciary marker locations in each of the 24 wells to determine the best focal plane. Variations in focal position primarily arise from substrate curvature during chip pressurization.

While the relative channel locations are known from the chip map, the positions of the trapped worms within the 40 parallel, 3 mm-long channels may vary depending on their size. To optimally center the 1.3 mm × 2.2 mm FOV to capture the entire body of as many worms as possible, a low-magnification overview image of the entire chip is first captured using a 2×, 0.08 NA objective. Then, all immobilized worms are detected using a trained YOLO object detection model [23], and the relative stage positions are calculated for the 5 FOVs needed to image the worms at 10×, 0.4 NA (8 × 150 μm spaced channels can fit in each 1.3 mm wide FOV) (**Supplementary Fig. 3C**). The system then automatically switches to the 10× objective and captures brightfield z-stack images using high-precision 3-axis translational stages. For each well, 5 FOVs are imaged, each comprising 18 z-planes (6 μm step size) and five timepoints in brightfield for volumetric timelapse acquisition (**Supplementary Fig. 3D**).

A single vivoChip experiment generates 12,984 images (~ 390 GB). Raw image data and associated metadata (timestamps, xyz coordinates, image capture and illumination parameters, treatment conditions etc.) are automatically transferred to a high-capacity network attached storage (NAS) server for preprocessing and downstream analysis.

### Image processing and phenotypic scoring

For each FOV with 8 channels, channel boundaries were identified by detecting fiduciary markers within each well and applying pre-defined internal channel spacing from the chip map. Channel coordinates were stored in the centralized database for subsequent multiparametric analysis.

Automated worm detection was performed using a deep learning–based method [46]. In brief, an image slice with optimal focus was identified from each z-stack by maximizing the variance of the Laplace-transformed image [54, 55]. An improved 2.5D U-Net architecture, including a classification head at the bottleneck, was trained, tested, and used to first classify each channel as full (entire worm within the FOV), partial (partial worm within the FOV), or none (no worm present) and then predict a segmentation mask for channels classified as full. For the new model, we expanded the training dataset to include small-sized worms close to the channel exit, decoupled the image-level classification and the pixel-wise segmentation models to facilitate independent optimization and model learning with 1 and 5 z-slice images around the best focus image, respectively. The segmentation mask, predicted by the new model, was used to calculate body length (via skeletonization of the mask), area (total segmented pixels), and volume (mask area integrated with channel heights) for each full worm.

Embryonic phenotyping was performed manually on all channels predicted as “full” by the ML model. All *in utero* embryos in these channels were scored and classified according to their developmental stage. Ten trained scorers independently scored and classified embryos using a custom graphical user interface (vivoAnalyzer, **Supplementary Fig. 4**). The software displays individual channels with zoom and z-plane navigation capabilities, allowing users to place a marker on each embryo and classify it as early- or late-stage embryo using the two-fold stage as the classification threshold. All automated image processing outputs (worm classification labels and segmentation masks) and manual annotations (marker positions and classifications of each embryo) were stored in the centralized database and exported for statistical analysis. The majority of embryo scoring was conducted in a blinded manner, with treatment conditions concealed from scorers.

### Statistical analysis

For DART data analysis, datasets were first cleaned to remove any worms for which the body segmentation mask failed, preventing dimensional calculations, or channels marked as unscorable by a user due to intestinal obstruction of the uterus (**Supplementary Fig. 5**). The remaining worms were then filtered to remove outliers in body volume or total embryo count (sum of early- and late-stage embryos). Outliers were identified using Tukey fences (1.5 × the interquartile range (IQR)) applied to each endpoint, and worms with measurements outside these fences were excluded. Inspection of excluded samples indicated that most were debris composed of shed worm cuticle and laid embryos clogging the channel. Mean phenotype scores were calculated per well and used to fit concentration-response curves from 3 biological replicates. One-way ANOVA was used to identify statistical differences between multiple well average values for different DMSO conditions.

To estimate repeatability, the coefficient of variation (CV% = ratio of standard deviation to mean) was calculated for every possible combination of 3 wells drawn from every corresponding well position across 5 independent biological replicates (10 combinations per well position, 24 well positions). The mean CV% and standard error of mean (SEM) across all 10 combinations were reported for each endpoint. To measure the strengths of the difference between 1% DMSO and methylmercury-treated worms, we calculated the Strictly Standardized Mean Difference (SSMD) for all 6 endpoints using 3 biological replicates. From all methylmercury conditions, we selected the lowest concentration to serve as a positive control for our case study that produced the SSMD ≥ 2 for all the DART parameters, indicating strong effects [56–59].

Assay power was estimated using the mean and standard deviations obtained from all 60 possible combinations (10 combinations per well position × 6 well positions with 1% DMSO) for all endpoints from repeatability experiments. Power calculations were performed for a fixed value of *n* = 3 technical replicates and a series of hypothetical treatment group means representing 2.5% – 50% effect sizes below the control means (one-sample, one-tailed t test, α = 0.05) using GraphPad Prism (version 10.6.1).

Concentration-response curves and effective concentration (EC_10_, EC_25_, and EC_50_) values were fitted using a 4-parameter logistic curve function using the “Find ECanything” nonlinear fit function of GraphPad Prism. The lower asymptote for each endpoint was constrained to the minimum biologically possible values of 0 embryos for Reproductive endpoints and estimated mean L1 body dimensions (length = 150 μm, area = 1,500 μm^2^, volume = 11,775 μm^3^) for developmental endpoints. For each EC_x_ estimate, the 95% confidence interval (CI) bands were calculated and plotted. Two concentration-response curves were compared using extra sum-of-square model to identify if the best fit values of EC_x_ values and Hillslope differ between two data sets. The p-value was denoted by ns (*p*-value ≥ 0.5), * (*p*-value ≤ 0.01), ** (*p*-value ≤ 0.001), *** (*p*-value ≤ 0.0001), and **** (*p*-value ≤ 0.0001).

## Results

### A robust C. elegans assay to study developmental and reproductive toxicity (DART)-related endpoints

**Figure 1:**
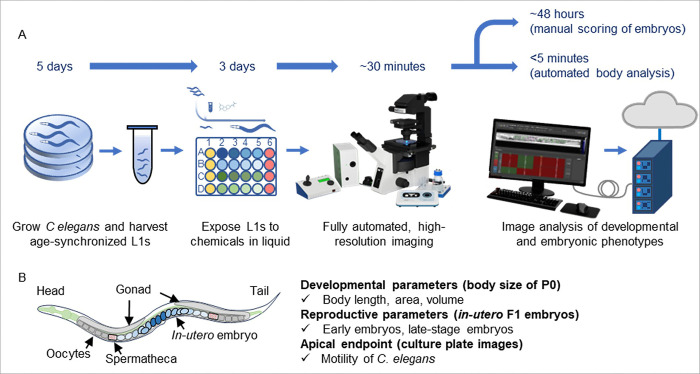
Schematic of the assay workflow for quantifying DART phenotypes in C. elegans following exposure to test substances. (A) Synchronized L1-stage C. elegans are cultured in liquid media in varying concentrations of a test substance or control chemical. After 72 hours of exposure, when control worms have reached D1 adult stage, worms are loaded into the vivoChip-24x microfluidic trapping and immobilization device for rapid, high-resolution 3D imaging. Software-assisted image analysis is then used to quantify embryonic phenotypes (manual scoring of ~30–40 embryos across ~1,000 worms, requiring ~48 hours) and developmental phenotypes (automated ML-based quantification of body dimensions completed in < 5 minutes) associated with DART toxicity. (B) Overview of DART and apical (worm motility) endpoints captured using vivoChip imaging data and plate images, respectively. In utero embryo number and developmental stage, indicated by different shades of blue objects, are quantified along with body developmental endpoints.

We developed a DART assay that quantifies *in utero* embryos within adult *C. elegans* at distinct developmental stages (early and late stages) following chemical exposure. *C. elegans* populations were exposed from the L1 stage for 72 hours and subsequently imaged using vivoChip devices to quantify embryo development relative to the 2-fold stage (Fig. 1A). Building on our previously established developmental toxicity (DevTox) platform, which uses ML-assisted image analysis [46] to rapidly quantify developmental endpoints in *C. elegans*, we extended this high-content imaging and analysis approach to capture reproductive phenotypes with high sensitivity. In addition to measuring worm body dimensions (length, area, volume) with high accuracy, we quantified the total number of embryos in each worm and classified each embryo by developmental stage (early- or late-stage embryo development). This approach of quantifying sub-lethal reproductive phenotypes enabled the determination of effective concentration (EC_50_) values with high confidence and low coefficient of variance (CV%).

The whole-organism 3D imaging enabled by vivoChip microfluidic platform provides sufficient resolution to determine the developmental stage of *in utero* embryos inside the intact *C. elegans* uterus [60]. The vivoChip image-based assay yields 6 DART-related endpoints: body length, body area, body volume, total embryos, early-stage embryos, and late-stage embryos (Fig. 1B). Additionally, brightfield imaging of culture plates prior to chip loading was used to confirm worm viability and motility. By directly quantifying *in utero* embryo number and classifying developmental progression during early adulthood, this assay captures sub-lethal reproductive toxicity while minimizing confounding effects from age-associated decline in the reproductive health and off-target effects such as altered egg-laying circuits. This DART platform facilitates rapid, high-content screening of chemicals and sensitive detection of sub-lethal developmental and reproductive perturbations.

### Automated high-resolution, whole-body imaging to extract DART endpoints

**Figure 2:**
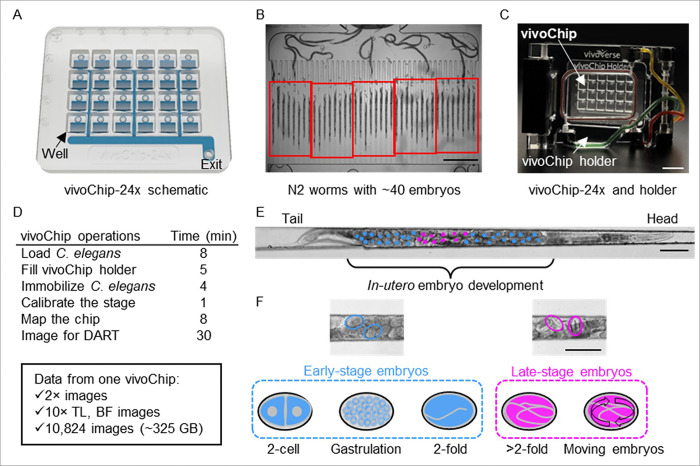
vivoChip-24x design and operation for high-resolution imaging in DART studies. (A) A schematic illustration of vivoChip-24x device containing 24 wells, each fluidically connected to 40 microfluidic trapping channels. (B) Representative image of 40 microfluidic channels under one of the 24 wells, containing N2 adult worms. Worms are trapped at different portions of the tapering channels according to body size. (C) The vivoChip-24x device mounted in its pressurized holder for controlled loading and immobilization. (D) Timeline and duration of each vivoChip operation workflow and total data volume produced from a single imaging experiment. (E)Representative 10× image of a trapped adult worm showing 31 early-stage embryos (blue) and 10 late-stage embryos (magenta). (F) Examples and schematics of embryos at developmental stages used for classification into early- and late-stage categories. Scale bars are 1 mm (B), 20 mm (C), and 100 μm (E and F).

The DART assay leverages the unique ability of vivoChip microfluidics platform to rapidly immobilize large numbers of *C. elegans* for high-resolution imaging of their entire body. Each vivoChip-24x device enables parallel immobilization up to 960 *C. elegans* (Fig. 2A), and the closely spaced microfluidic channels maximize information density in each FOV (Fig. 2B). A pressurized gasket system seals the vivoChip-24x device and applies a pre-defined pressure sequence for loading and immobilization of worms efficiently (Fig. 2C). Using automated stage navigation and objective switching, all wells are imaged sequentially, and complete volumetric datasets are acquired in ~ 30 minutes per chip.

High resolution 10× 0.4 NA brightfield z-stacks (18 planes at 6 μm spacing) captured the full uterus of each immobilized worm, enabling visualization of *in utero* embryos across developmental stages. This axial sampling accommodates minor variations in worm posture within the channels while preserving imaging speed. Prior to chip imaging, videos of the culture plates were collected to analyze worm motility (Fig. 2D).

Embryos were classified into two developmentally distinct categories: early-stage embryos (≤ 2-fold) and late-stage embryos (> 2-fold). The 2-fold stage provides a clear morphological boundary due to the rapid increase in structural complexity within the eggshell. Late-stage embryos often display fully formed larval structures, particularly in the central region of the uterus near the vulva, where embryos are frequently developed into young larvae. Visualization of individual larvae within the eggshell boundaries facilitates accurate classification. In some cases, movement within the eggshell was observable in time-lapse data (Figs. 2E–F), further supporting developmental staging. In future implementations, embryo motility could be incorporated as an additional quantitative endpoint.

### Image analysis pipeline for robust phenotyping

Imaging data were first pre-processed by identifying bounding boxes around individual channels, enabling analysis of each worm (**Supplementary Fig. 6A**). Downstream image analysis consisted of two separate processes; (1) automated quantification of worm body dimensions using a deep-learning-based segmentation (**Supplementary Figs. 6B-C**) and (2) semi-automated embryonic phenotyping using a GUI-based analysis software to streamline manual scoring (**Supplementary Figs. 6D-F**).

Previously, we developed a 2.5D UNET architecture for image-level classification and pixel-wise segmentation tasks [46]. Under this architecture, both tasks were jointly learned within a single network sharing a common encoder and bottleneck, trained using three z-slices around the best-focus image. While effective, this design introduced task coupling, limiting independent modification and optimization of the two objectives. To address this limitation, we decoupled classification and segmentation into separate models, each tailored to its respective task. The classification model comprises a convolutional encoder followed by a Vision Transformer (ViT) bottleneck and a classification head. The segmentation model consists of a convolutional encoder, a ViT-based bottleneck, and a convolutional decoder. Since image-level classification relies primarily on global morphological features, present in the best-focus image, we trained the classification model using a single best-focus image. In contrast, pixel-wise segmentation tasks rely on subtle changes in the image contrast to identify the object boundaries. To improve head and tail boundary detection, we fed a total of 5 z-stack images around the best-focus plane into the model. This approach captures features spanning multiple z-slices, improving boundary identification.

To further improve robustness under challenging imaging conditions, the training datasets for both models were expanded to include additional channels with smaller worms, located near the exit portion of the immobilization channel from both vivoChip-24x-L4 and D1 devices (**Supplementary Figs. 7**). For the classification model training, we generated ground truth data by classifying 5,305 channels as full, partial, or no worms class. For the segmentation model training, we used manually segmented masks from 4,360 channels with full worms. The classification model was evaluated on a test set of 538 images (441 full, 50 partial, and 47 channels with no worms) and achieved a weighted F1-score of 98.3%. The segmentation model was evaluated on a separate test set of 439 full worm images, achieving a mean Dice score of 98.5% (**Supplementary Fig. 8**). High prediction accuracy for both channel classification and worm segmentation was observed for worms immobilized in D1 (**Supplementary Figs. 9A-D**) and 4L (**Supplementary Figs. 9E-F**) devices. From segmented full-worm channels, we calculated body length (defined as the longest skeleton length) and body area. Body volume was then estimated by integrating the segmented area by the known channel height at each pixel. For each worm, these three body parameters were calculated, and population-level metrics were obtained by averaging values across worms within each well.

Embryo phenotyping is a challenging task, as each vivoChip-24x can immobilize up to 960 adult *C. elegans*, corresponding to approximately 40,000 *in utero* embryos requiring classification. Furthermore, the intertwined morphology of the uterus with the intestinal structures and gut granules requires 3D visualization of the worm uteri for accurate phenotyping. To facilitate robust embryo phenotyping, we developed a custom GUI-based software tool (vivoAnalyzer) that automatically displays each worm-containing channel in sequence, enables interactive zooming and z-stack navigation, and allows users to annotate the *xyz* centroid of each embryo while assigning early- or late-stage classification (**Supplementary Fig. 4**). When necessary, the user can use time-lapse frames to confirm late-stage embryos, exhibiting larval movement. Scoring was performed by 10 trained users after following validation of their scoring accuracy on a reference dataset. Worms were randomly assigned to users, and treatment conditions were blinded to minimize scoring bias.

### Repeatability of the DART assay

To evaluate the robustness and statistical reliability of the DART assay in predicting toxicity levels of substances, we studied repeatability across multiple independent experiments using the same test substances. For an assay to replace or augment existing DART methods, it needs to be scalable, reproduceable by different scientists using different batches of worms and capable of providing repeatable results over time.

**Figure 3:**
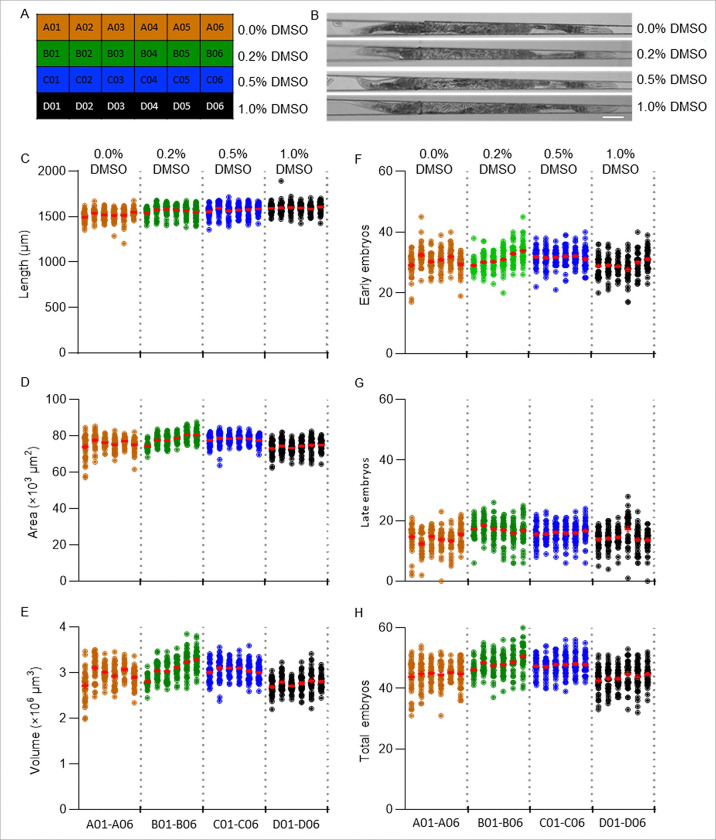
Representative DART parameters from a single biological replicate for different solvent concentrations. A total of 6 technical replicates were performed for each condition (one row per plate), and 5 biological replicates were performed on different days: results from one representative biological replicate is shown here. (A) Experimental design of a single biological replicate, containing 6 identical technical replicate wells for each 4 concentrations of DMSO. For each parameter, well averages were calculated Page 32/37 from up to 40 worms per well. (B) Example worm images from 0%, 0.2%, 0.5%, and 1% DMSO conditions. Scale bar is 100 μm. (C-H) Scatter plot of body length (C), body area (D), body volume (E), early-stage embryos (F), late-stage embryos (G), and total embryos (H) with each dot representing data from an individual worm for each well. The worms that passed the outlier filtering process for volume and total embryo for each well are presented in this plot. The mean ± SEM values for each well is represented in red. The vertical dashed lines separate the four DMSO conditions (A01-A06, B01-B06, C01-C06, and D01- D06).

We tested the repeatability of our DART assay by performing a series of 5 independent experiments (biological replicates) over two-month period using DMSO, a commonly used solvent in toxicity assays because of its wide applicability to a range of chemicals. Four DMSO concentrations (0.0, 0.2, 0.5, and 1.0%) were tested to characterize baseline phenotypes under solvent only control conditions. Each concentration was replicated across 6 wells (technical replicates) to assess well-to-well technical variability in addition to inter-experimental variability (Fig. 3A). We selected a 1% DMSO concentration as the solvent concentration because it allows us to dissolve the highest possible chemical concentrations near solubility limits while modestly increasing membrane permeability, which aids chemical uptake [12, 61, 62].

The worms developed into the D1 adult stage in all wells across all tested DMSO concentrations, including 1% DMSO (Fig. 3B). To examine variability across both biological and technical replicates, we compared all 30 well averages (5 biological replicates × 6 technical replicates) for all endpoints. For one representative biological replicate, the mean body parameters for 0% DMSO were 1,522 ± 8 μm (length, Fig. 3C and **Supplementary Fig. 10A**), 75.9 ± 0.6 × 10^3^ μm^2^ (area, Fig. 3D and **Supplementary Fig. 10B**), and 2.96 ± 0.06 × 10^6^ μm^3^ (volume, Fig. 3E and **Supplementary Fig. 10C**). For 1% DMSO, the corresponding values were 1,596 ± 3 μm (length, *p*-value < 0.0001), 74.2 ± 0.4 × 10^3^ μm^2^ (area, *p*-value = 0.19), and 2.76 ± 0.02 × 10^6^ μm^3^ (volume, *p*-value = 0.045). Embryo-related endpoints were similarly consistent. For 0% DMSO, worms exhibited 30.7 ± 0.6 early-stage embryos (Fig. 3F and **Supplementary Fig. 10D**), 14.0 ± 0.5 late-stage embryos (Fig. 3G and **Supplementary Fig. 10E**), and 44.7 ± 0.2 total embryos (Fig. 3H and **Supplementary Fig. 10F**), whereas for 1% DMSO, averages were 29.2 ± 0.5 (*p*-value = 0.23) early-stage embryos, 14.5 ± 0.6 (*p*-value = 0.86) late-stage embryos, and 43.7 ± 0.4 (*p*-value = 0.24) total embryos. Although, there were significant differences between 0% and 1% DMSO conditions for body length and volume, there were no significant differences between these two populations for body area, total embryos, early-stage embryos, or late-stage embryos (**Supplementary Fig. 10**).

Although body length and volume showed statistically significant changes (+ 4.9% and − 7.3%, respectively), the magnitudes of these differences were small. The well mean values obtained from all 30 wells from 5 biological replicates showed a similar trend among all 6 DART parameters (**Supplementary Fig. 11**). DMSO concentrations ≥ 1% has previously been reported to affect worm internal structure and posture, potentially causing mild changes in the body dimensions [33]. However, when comparing all 30 wells from the 5 biological replicates, we found no significant differences between 0% and 1% DMSO for early-stage, late-stage, and total embryo counts (**Supplementary Figs. 10D-F**).

**Figure 4.**
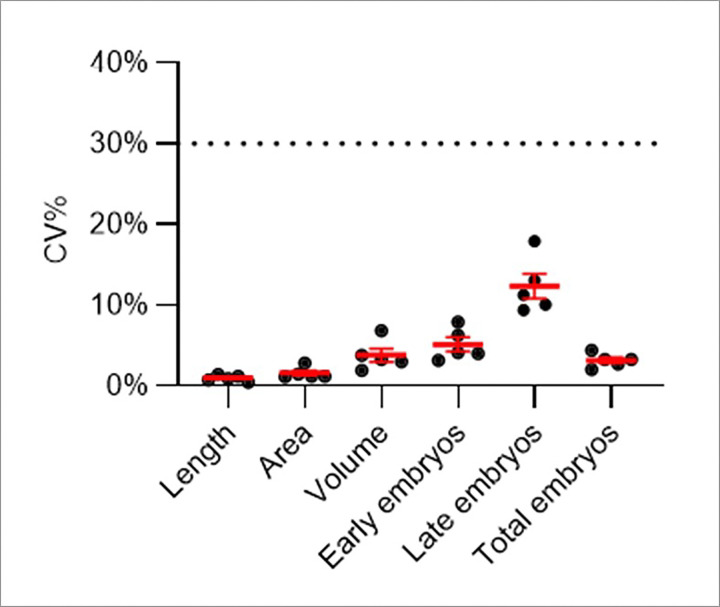
Coefficient of variation between technical replicate wells for 1% DMSO conditions. Scatter plot for the coefficient of variation (CV%) for 5 biological replicates for all 6 DART endpoints. Each data represents the CV% calculated using 6 technical replicates for 1% DMSO wells. The red line represents mean ± SEM. The dotted line represents the 30% CV line.

To test variability in the DART endpoints among technical replicates, we calculated coefficient of variation (CV%) between 6 technical replicates (Columns 1 to 6 in vivoChip-24x) for each condition. For 1% DMSO condition, the mean CV% values between the technical replicates were < 3.8% for developmental endpoints and < 12.5% for embryonic endpoints (Fig. 4). This data suggests high reproducibility among worm populations loaded and imaged in the vivoChip, confirming the absence of edge effects or location-based bias.

**Figure 5:**
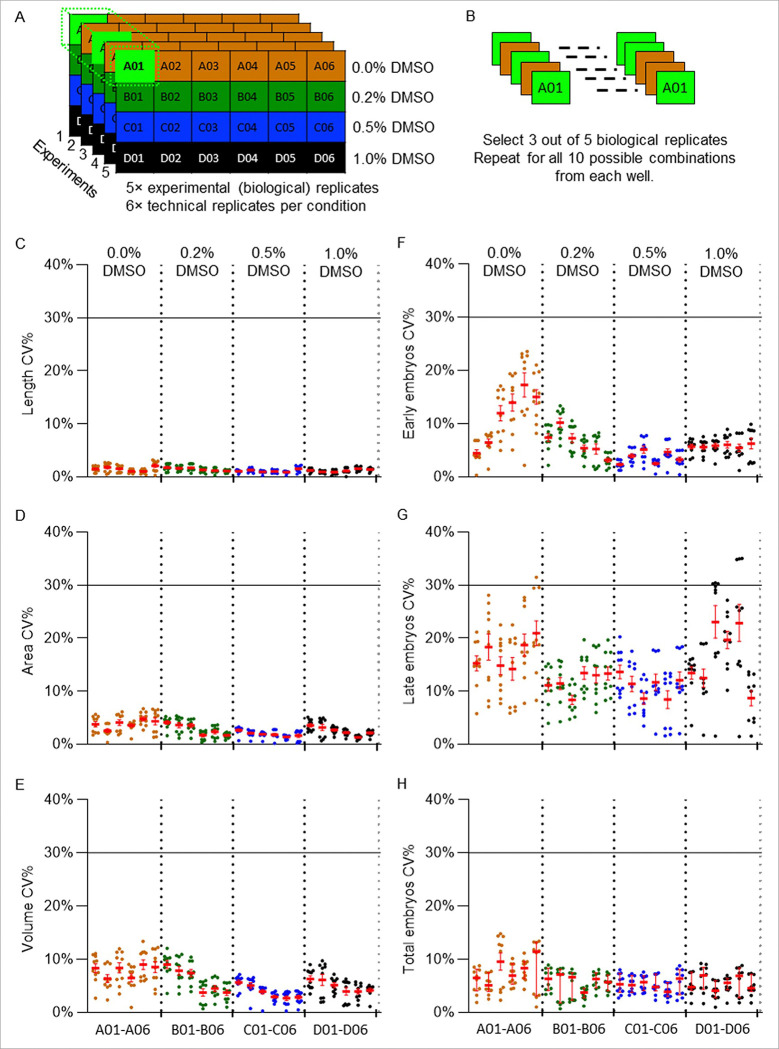
Repeatability of the DART assay across all five biological replicates for all six endpoints. (A) Experimental design consisting of 5 independent biological replicates, each containing 6 identical technical replicate wells for 4 concentrations of DMSO. (B) Schematic, illustrating how we calculated the coefficient of variation (CV%) values for all possible combinations of 3 corresponding wells from 5 experiments. Each well contained up to 40 worms. (C-H) CV% values body length (C), body area (D), Page 34/37 body volume (E), early-stage embryos (F), late-stage embryos (G), and total embryos (H). Each point represents the CV% derived from a unique combination of 3 corresponding wells across different experiments. The red horizontal line represents the mean ±SEM CV% values across all combinations for each DMSO concentration. The horizontal solid black line represents the 30% CV threshold. The vertical dashed lines separate the four DMSO conditions (A01-A06, B01-B06, C01-C06, and D01-D06).

To assess inter-experimental repeatability, we calculated the coefficient of variation (CV%) between 3 corresponding wells from 3 separate experiments (e.g. exp_1_ A01, exp_3_ A01, exp_5_ A01) and repeated this calculation for all 10 possible combinations of the 5 experiments (Figs. 5A-B). For the 0% DMSO populations, the mean CV% values across different experiments and 60 total combinations were 1.5 ± 0.1% for body length (Fig. 5C), 3.9 ± 0.2% for body area (Fig. 5D), 7.8 ± 0.4% for body volume (Fig. 5E), 11.5 ± 0.8% for early-stage embryos (Fig. 5F), 17.1 ± 0.9% for late-stage embryos (Fig. 5G), and 7.4 ± 0.5% for total embryos (Fig. 5H). For the 1% DMSO populations, the mean CV% were 1.2 ± 0.1% (length), 2.5 ± 0.2% (area), 5.0 ± 0.3% (volume), 5.8 ± 0.3% (early-stage embryos), 16.7 ± 1.1% (late-stage embryos), and 5.24 ± 0.3% (total embryos). All values were substantially below the 30% threshold considered acceptable under OECD test guideline #222 for earthworms [37, 63], indicating high inter-experimental repeatability of the assay.

### Statistical power of DART assay parameters

**Figure 6.**
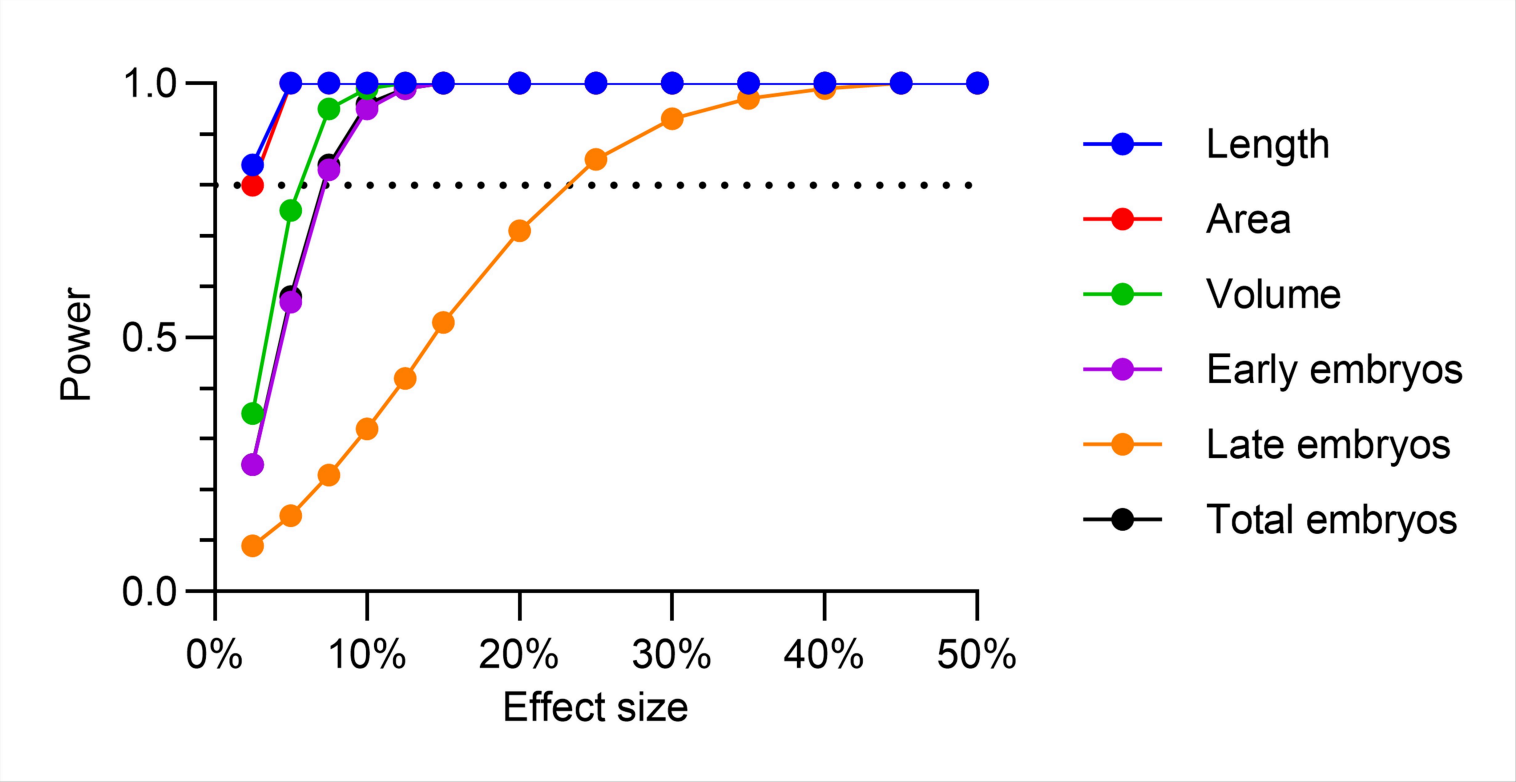
Statistical power for six DART parameters using 3 biological replicates. Predicted statistical power of the assay to detect different hypothetical changes in each phenotype relative to control (1% DMSO). Power calculations assume 3 biological replicates per condition, the mean and standard deviation measured from all 60 combinations of 3-replicate average values in control populations, and a one-sided significance level of α = 0.05. The dotted horizontal line indicates the 80% power threshold considered acceptable for a good assay.

To validate our DART assay design has sufficient statistical power of detecting small proportional changes relative to the control population using 3 biological replicates, we calculated the minimum detectable size effects with ≥ 80% power values. To estimate the power, we used the aggregated means and standard deviations from 60 possible combinations (10 combinations per well locations and 6 wells) and for all parameters measured in the 1% DMSO wells, that will serve as assay controls. With 3 experimental replicates, our assay achieved > 80% power to detect changes of ≥ 5.5% in developmental endpoints (body length, area, and volume) and ≥ 7% in total and early-stage embryos. For late-stage embryos, the minimum detectable effect size at the 80% power threshold was 23% (Fig. 6A), though increasing biological replicates would enable detection of lower reductions.

### DART assay with methylmercury

We next evaluated the assay using a well-studied toxicant, methylmercury, which has documented toxicity in *C. elegans* [20, 25, 64] and mammalian models [65]. The purpose of testing this chemical was to assess repeatability and precision in concentration-response studies, and to find the sensitivity of each endpoint. We ran 3 biological replicates spanning 20 concentrations of methylmercury between 0.0007 and 10 μM using the vivoChip-24x-D1 device.

Range-finding assays based on plate imaging showed that concentrations > 10 μM caused developmental arrest between the L1 and L3 stages, below the minimum size trappable in the D1 or L4 devices. Consistently, worms exposed to ≥ 8 μM developed slowly, remained approximately at the L4-stage, and slipped through the D1 chip channels due to their small size (**Supplementary Fig. 12**).

To enable imaging at higher concentrations, we ran a second set of experiments using the L4 devices, which can trap smaller worms down to early L4 stage. For the full concentration-dependent DART study, we used data from the D1 chip for lower concentrations and only used the data from L4 chips for the highest concentrations to complete concentration-response curves. Inclusion of the L4 chip data captured the 50% effect region for developmental endpoints and up to 100% effect region for embryo endpoints.

**Figure 7.**
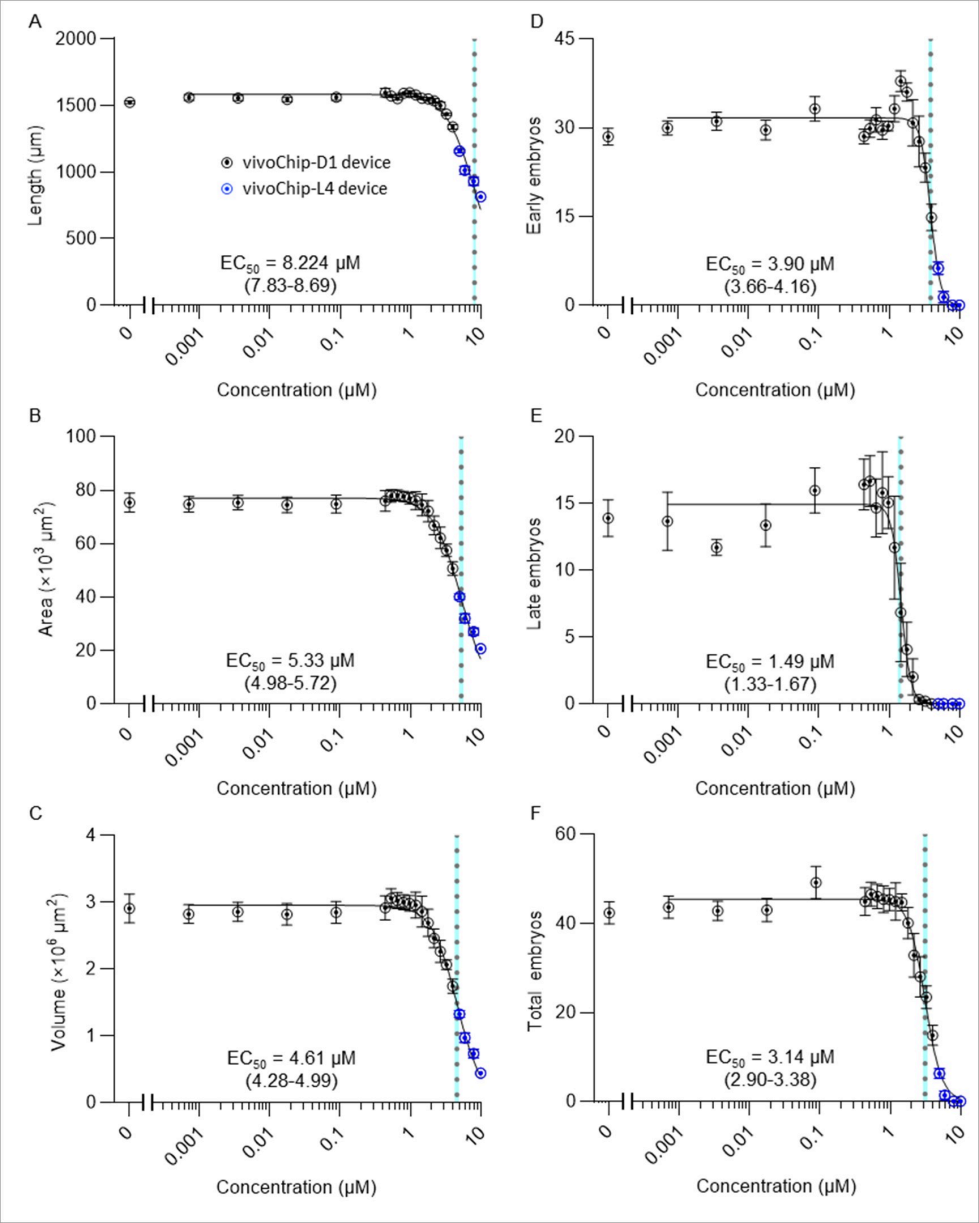
Concentration-response curves of six DART parameters following methylmercury exposure. The effects of methylmercury on (**A**) body length, (**B**) body area, (**C**) body volume, (**D**) early-stage embryos, (**E**) late-stage embryos, and (**F**) total embryos. Data for 0–4 μM methylmercury were obtained from 3 biological replicates performed using the D1 vivoChip devices (black symbols). Data for 5–10 μM methylmercury were obtained from 3 separate experiments performed using the L4 vivoChip devices (blue symbols), as worms at these concentrations were too small to be reliably trapped in the D1 devices. The combined well average values from both datasets were fitted with a 4-parameter logistic curve (solid black lines). EC_50_ estimates are indicated with dotted lines with corresponding 95% confidence interval bands shaded in light-blue color.

The combined results obtained from the D1 and L4 devices were fit with a 4-parameter logistic curve using the well averages after outlier filtering, and EC_10_, EC_25_, and EC_50_ values were estimated for each endpoint (Fig. 7 and **Supplementary Table 2**). For developmental endpoints, EC_50_ values (95% CI) were 8.22 μM (7.83–8.69) for body length (Fig. 7A), 5.33 μM (4.98–5.72) for body area (Fig. 7B), and 4.61 μM (4.28–4.99) for body volume (Fig. 7C), indicating that body volume is the most sensitive developmental metric. For the reproductive parameters, EC_50_ values (95% CI) were 3.90 μM (3.66–4.16) for early-stage embryos (Fig. 7D), 1.49 μM (1.33–1.67) for late-stage embryos (Fig. 7E), and 3.14 μM (2.90–3.38) for total embryos (Fig. 7F), indicating that embryo classification associated with different stages of development provides additional sensitivity and that late-stage embryos represent the most sensitive reproductive phenotype. Representative worm images are shown in **Supplementary Fig. 13**. Inter-experimental repeatability determined from the 1% DMSO control wells was high, with CV% values of 1.2% (length), 8.3% (area), 12.9% (volume), 8.8% (early-stage embryos), 17.1% (late-stage embryos), and 10.2% (total embryos), all well below the 30% threshold. In addition, the EC_50_ confidence intervals were narrow ( < ± 0.5 μM), further supporting good assay precision.

To study the effect of reducing the number of treatment conditions on EC_50_ values, we omitted every other methylmercury condition and reanalyzed the DART data from 10 out of the 20 conditions. We fitted the reduced 10-point dataset with a 4-parameter logistic curve and re-estimated EC_10_, EC_25_, and EC_50_ values (**Supplementary Table 3**). The resulting EC_50_ values were not significantly different from those obtained using the full dataset for the most sensitive developmental endpoint (body volume; EC_50_ = 4.51 μM; 95% CI: 4.03–5.08, *p*-value = 0.98, **Supplementary Fig. 14A**) and the most sensitive reproductive endpoint (late-stage embryos; EC_50_ = 1.51 μM; 95% CI: 1.26–1.80, *p*-value = 1.00, **Supplementary Fig. 14B**).

To identify a suitable concentration of methylmercury as a possible positive control for our DART assay, we calculated the SSMD for all 20 concentrations tested and all 6 DART parameters analyzed (**Supplementary Table 4**). SSMD provides a quantitative measure of assay quality by evaluating the separation between control and treated populations relative to variability. For 4 μM methylmercury, which produced a clear reduction in both developmental and reproductive parameters (**Supplementary Fig. 13**), SSMD values were: 5.18 (length), 3.24 (area), 2.77 (volume), 2.96 (late-stage embryos), 5.84 (early-stage embryos), and (total embryos). According to established criteria, SSMD values ≥ 3 indicate excellent assay quality, and values between 2 and 3 indicate good assay quality. Therefore, 4 μM methylmercury demonstrated good-to-excellent performance across all endpoints and was selected as a positive assay control concentration for the subsequent case study.

Based on the methylmercury concentration-response data, we further refined our DART assay design to test new chemicals at 10 concentrations per curve. This approach captures concentration-dependent effects across endpoints whose EC_50_ values may differ by up to 10 folds. Inclusion of sufficient concentration points is particularly important for endpoints with steep slopes, such as for late-stage embryos, the most sensitive parameter of our assay, to estimate precise EC_50_ values with narrow 95% CIs.

### Case study: DART assessment of an agrichemical

Following demonstration of assay precision and repeatability, we performed a concentration-response DART study using the triazole fungicide propiconazole [66–68], a chemical of interest to the agrichemical industry and ecotoxicology. First, we performed a preliminary range-finding assay by treating synchronized L1-stage N2 worms with 22 different concentrations of propiconazole ranging between 0.02–1,730 μM following our standard protocols. Concentrations > 334 μM were lethal (**Supplementary Fig. 15**).

Based on these results, we conducted a full concentration–response study with 10 propiconazole concentrations (0.08–334 μM), along with the solvent control (1% DMSO), and the positive control (4 μM methylmercury). Using 10 test concentrations and 2 controls (12 wells in total), concentration-response curves from 2 different chemicals can be tested on a single vivoChip-24x. At the highest propiconazole concentration (334 μM), worms exhibited delayed growth and were immobilized using the L4 chip.

**Figure 8.**
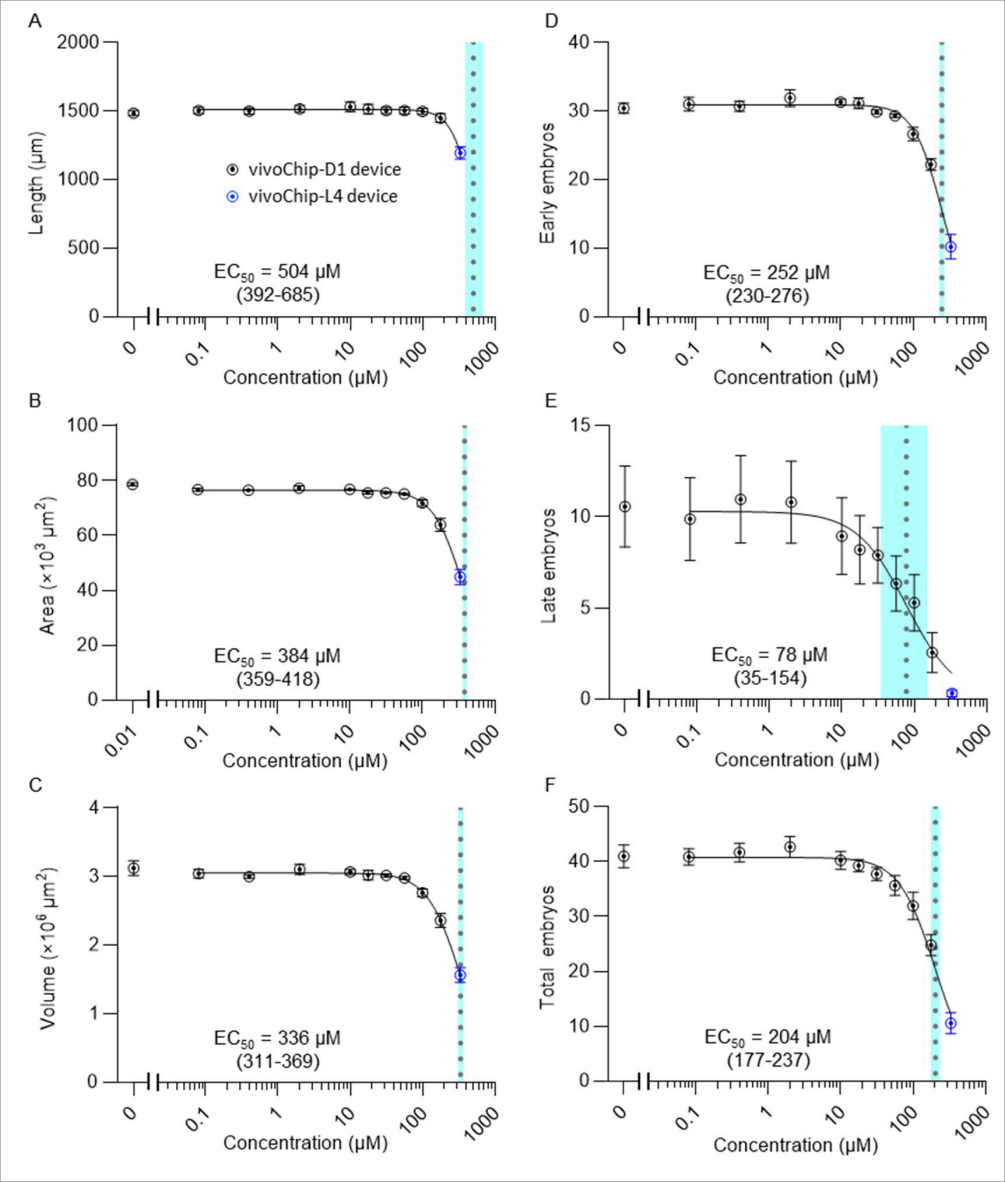
Concentration-response curves for DART parameters following propiconazole exposure. The effects of propiconazole on (**A**) body length, (**B**) body area, (**C**) body volume, (**D**) early-stage embryos, (**E**) late-stage embryos, and (**F**) total embryos. Data for 0–178 μM propiconazole were obtained from 3 biological replicates performed using the D1 vivoChip devices (black symbols). Data for 334 μM propiconazole were obtained from 3 separate experiments performed using the L4 vivoChips (blue symbols), as worms at this concentration were too small to be reliably trapped in the D1 devices. The concentration-response curves were fitted to the combined well-average values from both datasets with a 4-parameter logistic curve (solid black lines). The EC_50_ estimates are indicated with dotted lines, with corresponding 95% confidence interval bands shaded in light-blue color.

Similar to methylmercury, propiconazole produced distinct concentration-dependent effects (EC_50_ responses) across developmental (Figs. 8A–C) and reproductive (Figs. 8D–F) endpoints. Among developmental parameters, body volume exhibited a greater sensitivity (EC_50_ = 336 μM; 95% CI: 311–369; Fig. 8C) compared to body length (EC_50_ = 504 μM; 95% CI: 392–685; Fig. 8A). For reproductive endpoints, late-stage embryos were the most sensitive metric (EC_50_ = 78 μM; 95% CI: 35–154; Fig. 8E) with EC_50_ less than half that of total embryos (EC_50_ = 204 μM; 95% CI: 177–237; Fig. 8D). These results further support late-stage embryo phenotype as the most sensitive DART endpoint in this assay. The EC_10_, EC_25_, and EC_50_ values for 6 DART parameters are listed in the **Supplementary Table 4**, and representative worm images are shown in **Supplementary Fig. 16**.

### Comparative endpoint sensitivity analysis for DART assay

To further investigate the relative sensitivity and utility of different DART endpoints, we compared concentration-response curves normalized to baseline (no-effect) values for a widely used developmental parameter (worm body length), and total and late-stage embryo counts, for both methylmercury (Fig. 9A) and propiconazole (Fig. 9B). In addition, we quantified the percentage of worms that were alive, as identified to be moving in culture well plate images captured using 2× objective.

All worms remained alive at concentrations up to at least the highest concentration tested in the vivoChip concentration-response experiments. For example, 100% of worms in the plates were alive at 334 μM propiconazole and > 97% alive at 10 μM methylmercury, despite clear developmental impairment. These findings suggest that the observed responses were specifically attributable to DART-specific effects rather than lethality, an apical endpoint studied in *C. elegans*.

We calculated EC_10_ values for body length, total embryo, and late-stage embryo parameters for both methylmercury and propiconazole using the normalized concentration-response data (Fig. 9). The EC_10_ estimates, representing the onset of toxicity for each endpoint, were widely separated and followed the order: late-stage embryos EC_10_ < total embryos EC_10_ < body length EC_10_ < lethality onset. For methylmercury, the EC_10_ value for body length was 3.62 μM (95% CI: 3.33–0.92), total embryo was 1.94 μM (95% CI: 1.71–2.19), and late-stage embryo was 1.08 μM (95% CI: 0.92–1.24). For propiconazole, the EC_10_ value for body length was 256 μM (95% CI: 230–285), total embryo was 57 μM (95% CI: 43–73), and late-stage embryo was 14 μM (95% CI: 7–24). Notably, the EC_10_ for late-stage embryos preceded any measurable response in other endpoints, identifying late-stage embryo count as the most sensitive parameter.

For methylmercury and propiconazole, body length EC_10_ (3.62 μM and 256 μM), a commonly used developmental endpoint, was significantly different than the most sensitive late-stage embryo EC_10_ values (1.08 μM; *p*-value < 0.001 and 14 μM; *p*-value < 0.001) and were 3.4× and 18.3× higher, respectively. Viability remained high across the tested concentration ranges, confirming that the observed changes in each DART parameter represent bona fide developmental and reproductive effects rather than secondary consequences of lethality. Together, these findings demonstrate that the multiparametric DART assay provides independent and biologically meaningful assessments of developmental and reproductive endpoints without confounding effects from reduced viability.

This significantly lower toxicity threshold and large ratio of developmental and embryo parameters, especially for propiconazole, demonstrates that our multiparametric DART approach can detect reproductive toxicity independent of DevTox and viability. Also, the DevTox parameters are detected independent of viability. Such multiparametric analysis helps us to identify the adverse effects onset with potential benefits for prioritizing safety assessments of substances.

## Discussion

DART toxicity studies are required for hazard assessment of thousands of new and existing substances. However, high costs, long study durations, and a global push to reduce or eliminate mammalian models in routine testing present major bottlenecks. Here, we introduce a novel imaging-based DART assay using the model organism, *C. elegans*, which is faster, more cost-effective, and eliminates the need for vertebrate animals subject to welfare regulation.

Like human females, *C. elegans* has a complete reproductive system with conserved molecular pathways and several hallmarks of reproductive aging [40]. In certain toxicological domains, *C. elegans* have been shown to be as predictive as rodent models [13, 28, 43], supporting the translational relevance of the data obtained with this system for human reproductive toxicity assessment. While *C. elegans* has been used as a model organism in DART studies, previous approaches were limited by low throughput, labor-intensive methodologies, or reliance on relatively gross phenotypes such as brood size or body length [44, 45]. In this work, we developed a high-content, multiparametric imaging platform capable of detecting subtle multi-parametric DART phenotypes. This approach increases assay sensitivity to low-level exposures while improving throughputs, thereby addressing key limitations of existing DART testing paradigms.

We previously developed an ML-based imaging platform to assess DevTox using *C. elegans* as an alternative model organism [46]. This study expands upon that work by incorporating reproductive toxicity-related endpoints to assess multiple modes of action within a unified DART framework. We leveraged the vivoChip platform, which enables high-resolution, high-throughput imaging of 24 independent worm populations, with 40 worms per population (totaling 960 worms per vivoChip), while generating high-information-density datasets suitable for quantitative assessment of *in utero* embryo production and development progression. Two different vivoChip-24x designs, each optimized for trapping worms of different sizes, allowed us to analyze DART endpoint changes ranging from delayed L4s to normally developed adults. Integrating data obtained from both device configurations enabled comprehensive coverage across different sections of the concentration-response curve. This capability is particularly essential for multi-parametric assays, in which EC_50_ values for distinct endpoints may be widely separated, requiring adequate sampling across multiple exposure ranges to accurately resolve differential sensitivities.

For our DART assay, we exposed age-synchronized L1s for 72 hours and performed terminal imaging of *C. elegans* populations using the vivoChip platform. Worms were treated with multiple conditions, including solvent controls, in which the worms developed to the D1 adult stage during the exposure period. Because toxic chemical exposures can cause slow and asynchronous development, especially at high concentrations, it was necessary to start with precisely age-synchronized worms. Reproductive organ development in *C. elegans* spans from L1 to early adulthood. This process includes cell division of the two somatic gonad precursors and two primordial germ cells at the L1 stage, reorganization of the somatic gonad primordium during late L2, and formation of the sheath, spermatheca, and uterus during the L3 and L4 stages [41]. A 72-hour chronic exposure starting at the L1 stage maximizes the likelihood that chemicals reach the germline during critical windows of development. Such exposure may interfere with key biological events, such as mitotic proliferation, meiotic cell cycles, spermatogenesis, oogenesis, oocyte maturation, ovulation, and the entire course of *in utero* embryogenesis up to the start of neuromuscular movement within the egg [69, 70]. Imaging all treatment groups in an experiment within a narrow time interval further ensured accurate comparisons across concentrations and minimized variability introduced by developmental timing differences.

To capture adverse effects on reproduction, we identified and quantified all embryos within the uterus of individual *C. elegans* hermaphrodites and classified them as early- or late-stage embryos based on distinct morphological features, using the 2-fold stage as the classification threshold. We also calculated total embryo counts by adding early- and late-stage embryos for each worm. While the total embryo count serves as an *in utero* proxy for the traditional brood size metric, the late-stage embryo counts provide an indicator of successful embryonic development, correlating with the hatching efficiency measurements. Compared to traditional reproductive toxicity assays, our assay offers several advantages, including (1) scalability enabled by well-plate-compatible liquid culture, (2) simultaneous testing of 24 independent populations, (3) high-resolution imaging of up to 40 *C. elegans* per population to quantify *in utero* embryonic development, and (4) single time point imaging, which reduces assay complexity while preserving multiparametric information content.

Traditional brood size assays performed using NGM plates are labor-intensive and low-throughput [44]. Although microfluidic-based automated egg counting systems have been developed [49–51], these platforms are technically complex, require continuous imaging over 3–6 days, and consume large amounts of test substances due to constant perfusion. In contrast, our DART assay quantifies and classifies all *in utero* embryos within hermaphrodites on the first day of adulthood, when embryo production enters the peak phase. Assessing DART endpoints at this early adult stage offers several key advantages: (1) it enables scoring of high-quality embryos while avoiding confounding effects from age-related gonadal atrophy; (2) embryos are typically aligned in a single row, reducing phenotyping errors that arise from multilayered stacking in older worms; (3) the readout is independent of the egg-laying circuit, minimizing data drift caused by off-target neuromuscular effects; and (4) the assay is based on direct morphological features of embryogenesis rather than late-stage neuromuscular strength, which influences larval hatching outcomes. In addition, our DART assay relies on image analysis and scoring of brightfield images of wild-type worms and does not require any reporter strain or fluorescent biomarkers. This aspect facilitates application of our DART assay to large numbers of wild isolates with diverse genetic backgrounds, enabling characterization of genetic susceptibility to environmental toxicants using naturally occurring variation in *C. elegans* populations [22].

For any newly developed method, intra-laboratory repeatability (and ultimately inter-laboratory reproducibility) is vital for consideration by regulatory bodies [10, 71]. To address repeatability, we established robust and detailed protocols for *C. elegans* husbandry, synchronization, chemical exposure, and culture conditions to minimize inter-experimental variability. Our DART assay is highly data-intensive, generating ~ 11,000 images and nearly ~ 325 GB of data in less than 30 minutes from a single experiment. To enable even higher throughput, we developed a minimal data acquisition protocol to reduce data volume while preserving the ability to produce all the DART data presented in this study. This optimized approach, suitable for large-scale studies, collects 2,184 images (~ 66 GB) from a single vivoChip by limiting imaging data from a single timepoint, 18 z-slice brightfield images. To manage such large volumes of data and support multiparametric phenotypic analysis, we developed the vivoScreen platform, which integrates reliable hardware components with rigorously validated and tested software, a centralized database infrastructure, and both local and cloud-based data storage systems. Every experiment is archived with comprehensive metadata, including experimental details, imaging conditions, analysis outputs, and final reports, ensuring full traceability. Despite the large data volume, image analysis is performed efficiently using an ML-based pipeline for DevTox parameters with an accuracy that is comparable to human scorers while requiring only a fraction of the time. Embryo phenotypes are then scored using semi-automated, user-friendly software designed to streamline manual embryo annotation and improve scoring consistency.

The mean coefficient of variation (CV%) for 1% DMSO ranged from 1–5% for developmental endpoints and 6–17% for embryo-related endpoints, values well below thresholds considered acceptable for regulatory-approved assays, including those described for earthworms [37]. This low variability confers high statistical power, even with relatively few experimental replicates. Power analysis indicates that 3 independent experimental replicates are sufficient to detect > 23% changes across all endpoints, while smaller effect sizes can be reliably identified with additional replicates. While the late-stage embryo phenotype exhibited the highest variability, it was also the most sensitive endpoint in terms of EC_50_. The higher variation likely reflects the oscillatory pattern of egg laying, as embryos are laid in clusters every 1–2 hours [72]. Depending on timing, worms may be immobilized and imaged immediately before or after an egg-laying event, introducing natural biological variability into embryo counts. In addition, we confirmed that 1% DMSO, used as the solvent control, does not contribute any significant adverse effects on embryo parameters, enabling testing at relatively high treatment concentrations of many chemicals [61, 62].

We validated the DART assay using two reference chemicals: methylmercury and propiconazole. Methylmercury was chosen because of its extensive evaluation across variety of species and toxicology domains, including DART, neurotoxicity, metabolism, gene expression, stress response, and cell division [20, 25, 73–76]. Propiconazole was chosen as it is a widely used fungicide with well-characterized toxicology profiles in mammals and other model organisms [66, 67, 77, 78]. Both chemicals showed concentration-dependent effects on all 6 endpoints studied within our DART assay. These endpoints exhibited differential sensitivities based on EC_50_ values, with body volume emerging as the most sensitive developmental parameter and late-stage embryos as the most sensitive reproductive parameter. We observed narrow 95% confidence interval bands and low CV% values for the methylmercury control wells, indicating high assay precision. The assay quality, measured using SSMD, was good for ≥ 4 μM methylmercury, supporting its suitability as a positive assay control for all endpoints.

The onset of DART toxicity for methylmercury in our assays, based on the late-stage embryo endpoint, occurred at ~ 1 μM, consistent with previously reported *C. elegans* DART studies [20]. For propiconazole, adverse effects on fathead minnow fecundity and *Daphnia* embryonic development have been reported at 1.46 μM (0.5 mg/L), which is within an order of magnitude of our EC_10_ value of 14 μM [77, 78]. A recent study in zebrafish reported a DART EC_50_ of a 1.62 mg/L, (~ 2 μM propiconazole equivalent) for a commercial formulation, and additional studies have observed developmental abnormalities at 0.73–1 μM [79–81]. These comparisons across different species could be due to differences in chemical uptake or accumulation, which can be addressed by measuring tissue-level concentration using analytical methods. In *C. elegans*, a developmental toxicity assay and a brood-size-based reproductive toxicity assay reported an EC_50_ of 223–249 μM and 141 μM, respectively [82, 83]. Using the most sensitive late-stage embryos parameter in our assay, these previously reported values are ~ 1.8–3.2× higher than the EC_50_ value of 78 μM observed in our assay. These differences could be associated with the differences in the food source, DMSO concentration, exposure duration, and endpoint definition. While absolute effective concentrations of propiconazole in our results are higher than those reported in vertebrate systems, the *C. elegans*-based DART platform offers substantial advantages in cost, scalability, and throughput. Compared with rodent colonies and fish aquaria, this approach requires significantly lower resources and eliminates labor-intensive and subjective phenotyping of complex reproductive phenotypes on an animal-by-animal basis. Moreover, imaging-based multiparametric design enables objective and quantitative assessment of developmental and reproductive phenotypes.

All endpoints studied in our assay are biologically interlinked, and reproductive (embryo) phenotypes may, in part, be affected by developmental perturbations. However, the effective concentrations for the different endpoints were clearly separated, suggesting distinct sensitivities rather than a uniform secondary response. Total embryo numbers declined at lower concentrations than those required to reduce body size, indicating that the germline is specifically affected and that the observed reproductive effects are not simply a consequence of delayed general development. Moreover, reductions in late-stage embryos occurred before a decrease in total embryo counts, consistent with direct impairment of embryonic development rather than a simple reduction in embryo production. Importantly, the worms remained > 97% viable and motile across all tested concentrations. Therefore, the measured effects reflect DART responses rather than the apical endpoints such as lethality.

The novel DART platform we have developed is both faster and more cost-effective than traditional vertebrate-based assays. Thanks to the short life cycle of *C. elegans*, culture and treatment durations are measured in days, while chip loading and imaging times are < 30 min per experiment. In addition to improved efficiency, the assay eliminates the need for vertebrate animals subject to welfare regulations. Data analysis can also be performed rapidly. The ML inference pipeline used for body-dimension quantification can process a single chip (~ 1,000 *C. elegans*) in < 5 minutes using standard desktop PCs. Comparable processing times are expected for automated embryo detection once fully optimized ML-based models are implemented. Across these studies, we collected and annotated data from over ~ 389,000 embryos across ~ 9,200 worms under various treatment conditions, including detailed classification of developmental stage and extraction of *xyz* centroid coordinates within image stacks. This extensive dataset provides a strong foundation for training next-generation ML models to fully automate embryo detection and staging. We are currently developing new ML models that will enable fully automated phenotyping of all DART endpoints within very short processing times, eliminating the current time-intensive manual analysis and substantially increasing scalability.

This study demonstrates the sensitivity, repeatability, and precision of our DART assay. The implementation of automated embryo scoring will further enable high-throughput screening, allowing rapid and cost-effective testing of large chemical libraries for hazard prioritization and comparative safety assessment within defined chemical spaces.

## Supplementary Material

Supplementary Files

This is a list of supplementary files associated with this preprint. Click to download.

• vivoDARTPaperSupp.pdf

## Data Availability

The datasets generated and/or analyzed during the current study are available from the corresponding authors upon reasonable request.
